# Correlation between the thyrotropin-thyroxine resistance index and insulin resistance in polycystic ovary syndrome patients with phlegm-dampness syndrome

**DOI:** 10.3389/fendo.2026.1940904

**Published:** 2026-10-08

**Authors:** Ying Liu, Ming Zhang, Ying Wang, Xueyan Lv, Shuang Ma, Jinglu Yu, Hongying Kuang, Lihui Hou, Qingxia Song

**Affiliations:** 1 Heilongjiang University of Chinese Medicine, Harbin, China; 2 The First Affiliated Hospital of Heilongjiang University of Chinese Medicine, Harbin, China; 3 Affiliated Hospital of Shandong University of Chinese Medicine, Jinan, China; 4 Suzhou Hospital of Traditional Chinese Medicine Affiliated to Nanjing University of Chinese Medicine, Suzhou, China

**Keywords:** correlation analysis, insulin resistance, metabolic disorders, phlegm-dampness syndrome, polycystic ovary syndrome, subclinical hypothyroidism, thyroid hormone sensitivity, TT4RI

## Abstract

**Introduction:**

Polycystic ovary syndrome (PCOS) patients classified as the phlegm-dampness syndrome typically suffer from metabolic dysfunction mainly driven by insulin resistance (IR). Indices evaluating thyroid hormone responsiveness have emerged as promising biomarkers for metabolic disease research. However, limited research has investigated the thyrotropin-thyroxine resistance index (TT4RI), a marker quantifying central pituitary thyroid sensitivity, in this distinct PCOS subgroup. This study aimed to explore the association between TT4RI and IR in PCOS patients with phlegm-dampness syndrome.

**Methods:**

We enrolled 486 PCOS patients with complete baseline data in the present study. Among them, 264 patients were diagnosed with PCOS accompanied by phlegm-dampness syndrome. The 264 patients were further subdivided into two subgroups: with IR (n=208) and without IR (n=56), and clinical indicators were compared between the two subgroups. We evenly stratified the 264 phlegm-dampness syndrome PCOS patients into three groups to perform intergroup comparisons. Spearman’s correlation and multivariate linear regression analyses were used to investigate the association. ROC curves were also employed to compare the discriminatory performance of TT4RI, thyroid-stimulating hormone index(TSHI), and FT3/FT4 in identifying insulin resistance in these patients(n=264). The sole primary outcome of this study was HOMA-IR.

**Results:**

Relative to the subgroup without IR (n=56), the subgroup with IR (n=208) patients displayed significantly higher blood pressure, obesity-related anthropometric values, triglycerides (TG), fasting plasma glucose (FPG), fasting insulin (FINS), homeostasis model assessment of insulin resistance (HOMA-IR),free triiodothyronine (FT3), thyroid-stimulating hormone (TSH) and TT4RI (P<0.05).Patients in the high TT4RI tertile group exhibited a relatively more prominent phenotype of IR. TT4RI exhibited positive correlations with weight, body mass index (BMI), FPG, FINS, HOMA-IR, free thyroxine (FT4), and TSH (P<0.05).In the multiple linear regression model, after adjusting for age, BMI, systolic blood pressure(SBP) and SHBG, only a marginally significant association was observed between HTT4RI group and IR. ROC analysis indicates that TT4RI exhibits limited discriminatory performance for identifying IR among PCOS patients with phlegm-dampness syndrome.

**Conclusions:**

Among phlegm-dampness syndrome PCOS women, diminished central pituitary thyroid hormone sensitivity is associated with increased insulin resistance. Further prospective research is needed to confirm these findings and explore clinical relevance.

## 1 Introduction

Polycystic ovary syndrome (PCOS) is a reproductive endocrine disorder that predominantly affects women, characterized by oligomenorrhea, clinical or biochemical hyperandrogenism, and polycystic ovarian morphology. Furthermore, it frequently coexists with insulin resistance (IR), abdominal obesity, and abnormalities in the metabolism of fats and carbohydrates (1). Although the exact cause of PCOS is still unknown, insulin resistance and compensatory hyperinsulinemia are thought to be the main factors influencing its pathophysiology (2). When diagnosing PCOS according to Traditional Chinese Medicine patterns based on its clinical manifestations, it is often classified under syndromes such as “delayed menstruation” and “infertility”, and treatment typically focuses on the liver, spleen, and kidney. Among the clinical patterns of PCOS, the “phlegm-dampness” syndrome is the most prevalent and is associated with more severe metabolic disturbances (3).

The thyroid is the most significant endocrine organ in the body; it controls both female reproductive processes and metabolic rate. Studies have shown that thyroid dysfunction often leads to menstrual disorders, infertility, and metabolic disorders in women with PCOS (4). Thyroid function is influenced by multiple components of the hypothalamic-pituitary-thyroid (HPT) axis, and evaluation based on a single parameter may have limitations. Based on this, this study introduces the concept of the thyroid hormone sensitivity index. Describing the body’s responsiveness to thyroid hormones, thyroid hormone sensitivity has drawn rising research interest for its potential links to metabolic disorders such as type 2 diabetes mellitus. Recent evidence suggests that diminished thyroid hormone sensitivity may constitute a risk factor for metabolic diseases (5–7). Several observational clinical studies have confirmed that the prevalence of thyroid disease among women with PCOS is significantly higher than that in age-matched control groups (8, 9). However, few clinical studies have investigated the association between the thyroid hormone sensitivity index and PCOS with phlegm-dampness syndrome. Therefore, this study aims to explore the correlation between the thyrotropin-thyroxine resistance index (TT4RI) and insulin resistance in a population of PCOS patients with phlegm-dampness syndrome, with the hope of providing a novel endocrine perspective on the pathological mechanism of insulin resistance in PCOS patients with phlegm-dampness syndrome.

## 2 Materials and methods

### 2.1 Study population

We retrospectively screened medical records of 493 patients with PCOS who were diagnosed according to standard protocols at the Gynecology Outpatient Clinic of the First Affiliated Hospital of Heilongjiang University of Traditional Chinese Medicine between February 2023 and September 2025.Seven cases with missing key variables, which including TT4RI, homeostasis model assessment of insulin resistance(HOMA-IR), age, body mass index (BMI), systolic blood pressure(SBP),sex hormone-binding globulin(SHBG) and phlegm-dampness syndrome information) were excluded. Finally, 486 PCOS patients with complete baseline data were enrolled in the present study. The flow diagram for this retrospective analysis is shown in **Figure 1**. This study was approved by the hospital’s Ethics Committee (Approval No.: HZYLLKY201800601) and complies with the Declaration of Helsinki.

**Figure 1:**
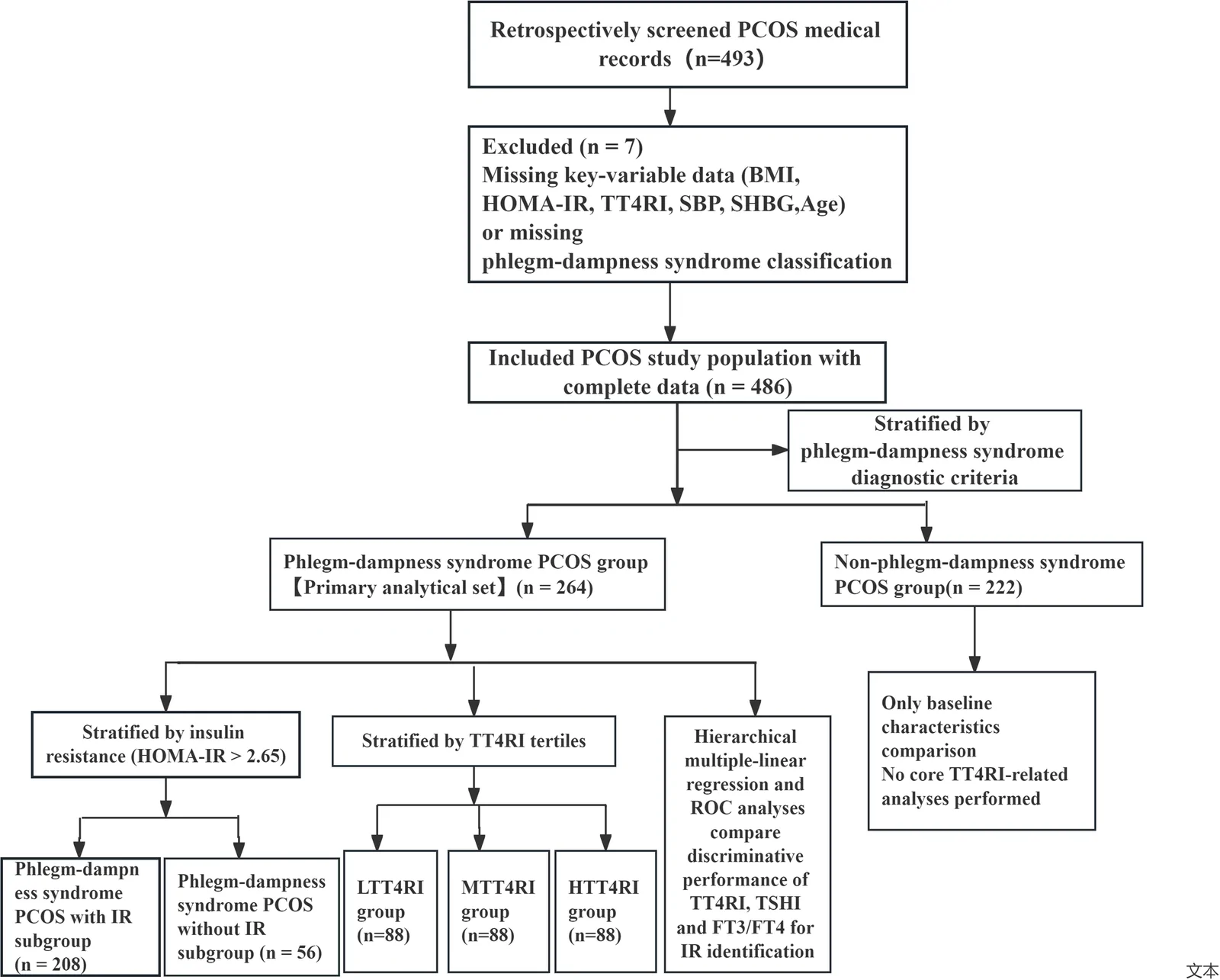
Flowchart for case-data screening and grouping of the retrospective analysis).STROBE-compliant flow diagram showing patients screening, exclusion, inclusion (n=486), grouping by phlegm-dampness syndrome, and subsequent stratification by insulin resistance and TT4RI tertiles for the retrospective PCOS study.

### 2.2 Diagnostic criteria

Referring to the 2018 edition of the “*Chinese Guidelines for the Diagnosis and Treatment of Polycystic Ovary Syndrome*” (10), the core diagnostic symptoms are oligomenorrhea, amenorrhea, or irregular uterine bleeding. Auxiliary diagnostic criteria require fulfillment of any one of the following:①clinical manifestations associated with hyperandrogenism or laboratory tests indicating hyperandrogenism;② Pelvic ultrasonography revealing polycystic ovarian morphology.

Based on the State Administration of Traditional Chinese Medicine’s “*Clinical Pathways for Traditional Chinese Medicine: 105 Diseases Across 24 Specialties (Trial Version)*” (11), the criteria are as follows: ① Primary symptoms: prolonged infertility despite marriage, delayed menstruation, or even amenorrhea, obesity; ② Secondary symptoms: profuse vaginal discharge, white in color and odorless; physical lethargy, fatigue, heaviness, and drowsiness; scanty menstrual flow or pale-dark menstrual blood; fullness and distension in the chest and abdomen; a pale, swollen tongue or one with tooth marks on the edges, with a white or greasy coating; a slippery or deep-thready, deep-slow pulse. A diagnosis can be made if one primary symptom and three or more secondary symptoms are present. Cases with mixed or other patterns are comprehensively diagnosed as non-phlegm-dampness syndrome. This scale is routinely used in outpatient clinical practice. Two TCM physicians independently performed syndrome differentiation with standardized training in gynecological pattern differentiation. A senior chief physician adjudicated any disagreements between the two physicians. This study retrospectively analyzed archived medical records. Since only the final consensus diagnostic result was preserved in the medical records, we were unable to obtain two sets of independent original judgments to calculate Cohen’s Kappa for inter-rater agreement. This is recognized as a limitation of this study.

### 2.3 Inclusion and exclusion criteria

#### 2.3.1 Inclusion criteria

① Patients met the above-mentioned diagnostic criteria for PCOS; ② Complete medical records containing archived TCM syndrome scale records for phlegm-dampness syndrome differentiation were available.

#### 2.3.2 Exclusion criteria

① Patients with pituitary amenorrhea or other conditions that prevent participation in the study; ② Patients who have participated in other clinical studies within the past month; ③ Patients with congenital malformations of the reproductive organs or menstrual abnormalities caused by organic lesions; ④ Patients with other conditions associated with excess androgens, such as androgen-secreting ovarian or adrenal tumors, Cushing’s syndrome, etc.; ⑤ Patients with organic thyroid diseases (hyperthyroidism, hypothyroidism, thyroiditis, etc.); ⑥ Use of medications interfering with endocrine and metabolic profiles within the past 3 months, including thyroid-related medications, sex hormone-related preparations, insulin sensitizers, antidiabetic and lipid-lowering drugs; ⑦ Incomplete clinical or laboratory data.

### 2.4 Observation criteria

Collect and organize the required clinical data through the hospital’s electronic medical record system. The main content is as follows:

#### 2.4.1 Collection of general data record

Patients’ baseline clinical information, including age, blood pressure, height, body weight, and waist circumference(WC), while also completing modified Ferriman-Gallwey score and acanthosis nigricans score, and BMI was calculated. The formula was as follows: 


$$BMI=body\;weight\;(kg)/height^{2}\;(m^{2})$$

#### 2.4.2 Collection of serum parameters

All serum indices were determined in the clinical laboratory of our hospital. (1) Glucose and lipid metabolism markers: Patients were instructed to maintain a light diet and undergo blood sampling after overnight fasting, including fasting plasma glucose (FPG), fasting insulin (FINS), and triglycerides (TG).HOMA-IR and HOMA-β were calculated. The HOMA-IR value > 2.65 confirms a diagnosis of insulin resistance (12). The formulas were as follows:


(HOMA−IR)=FPG(mmol/L)×FINS(μU/mL)/22.5;(HOMA−β)=20×FINS/(FPG–3.5)

(2) Sex hormone markers: Samples were collected from patients in the morning during a specific period (days 2–5 of the natural menstrual cycle or withdrawal bleeding). The experiment employed chemiluminescence technology to quantitatively measure serum levels of luteinizing hormone (LH), follicle-stimulating hormone (FSH), testosterone (T), dehydroepiandrosterone sulfate (DHEAS), and SHBG.

(3) Thyroid hormone markers: Test the following three serum markers: free triiodothyronine(FT_3_), thyroid-stimulating hormone (TSH), and free thyroxine(FT4), and calculate the FT_3_/FT_4_ ratio, TSHI, and TT4RI based on the test results. The formulas are as follows:


TT4RI=FT4 (pmol/L)×TSH (mIU/L);TSHI=ln(TSH (mIU/L))+0.1345×FT4(pmol/L)

FPG and TG were measured using an automatic biochemical analyzer (Model: LABOSPECT 008α, Manufacturer: HITACHI) and matched commercial kits(Kit manufacturer: HITACHI Laboratories). Other serum markers were detected by an automatic chemiluminescence analyzer (Model: Alinity i, Manufacturer: Abbott) and matched commercial kits (Kit manufacturer: Abbott Laboratories). The intra-assay and inter-assay coefficients of variation, as well as laboratory reference ranges for all serological indicators, are summarized in **Supplementary Table S1**. For some indicators, intra-assay and inter-assay coefficients of variation records were not archived in laboratory files and are marked as “Not archived” in the **Supplementary Table**. All laboratory measurements were performed under standard quality-control conditions.

### 2.5 Statistical methods

Data analysis in this study was performed using SPSS 27.0. Statistical descriptions of categorical data were presented as sample size (n) plus percentage (%), and differences between groups were compared using the chi-square test. Quantitative data that followed a normal distribution were expressed as mean ± standard deviation (x̄ ± s). When variances were homogeneous, an independent-samples t-test was used for comparisons between two groups; for multiple groups, one-way analysis of variance (ANOVA) was performed first, followed by LSD t-tests for intergroup comparisons. Furthermore, for data that did not follow a normal distribution or had unequal variances, the median (Q1, Q3) was used. Comparisons between two groups were performed using the rank-sum test, while comparisons among multiple groups were performed using the Kruskal-Wallis test, followed by Dunn-Bonferroni pairwise comparisons (with Bonferroni correction for pairwise tests within each individual variable). Spearman’s correlation analysis was used to investigate the correlation between the TT4RI index and various clinical indicators of PCOS with phlegm-dampness syndrome.

In this study, HOMA-IR was defined as the primary outcome variable, The P-value for HOMA-IR was derived from the unadjusted primary analysis. Other anthropometric, metabolic, reproductive, and thyroid-related variables were set as secondary exploratory outcomes, which should only serve as preliminary clues for future research. The Benjamini-Hochberg false discovery rate (FDR) method was applied for multiple-comparison correction of secondary outcomes in **Tables 1**–**3** to control type I error. The P-value < 0.05 was considered statistically significant.

**Table 1:** Baseline characteristics of phlegm-dampness syndrome PCOS patients with and without IR.

Variable	Phlegm-dampness syndrome PCOS with IR (n=208)	Phlegm-dampness syndrome PCOS without IR (n=56)	*t/z*	*P*
n	208	56	-	-
Age (years)	24.00 (18.00,29.00)	26.00 (20.75, 29.00)	-1.163	0.268
SBP (mmHg)	119.00 (112.00,131.00)	109.00 (100.00,113.00)	-5.800	0.002
DBP (mmHg)	81.00 (73.75,89.00)	75.00 (69.00,81.00)	-3.624	0.002
Weight (kg)	80.00 (72.50, 88.50)	66.00 (58.00,71.38)	-6.820	0.002
BMI (kg/m^2^)	29.88 (27.29,31.91)	24.94 (21.88,27.22)	-6.805	0.002
WC (cm)	95.49 ± 11.27	84.67 ± 12.11	6.275	0.002
mFG score	5.50 (3.00, 9.00)	4.00 (2.00, 6.00)	-2.813	0.009
Acanthosis nigricans score	1 (0,2)	0 (0,1)	-4.916	0.002
FSH (mIU/mL)	4.68 (3.73,5.44)	4.71 (3.68,5.58)	-0.413	0.680
LH (mIU/mL)	7.15 (5.16,9.37)	8.69 (4.91, 11.33)	-1.892	0.075
T (nmol/L)	1.91 (1.45, 2.31)	1.76 (1.35, 2.18)	-1.581	0.138
DHEAS (μmol/L)	8.00 ± 3.49	8.72 ± 3.04	-1.401	0.186
SHBG (nmol/L)	18.60 (13.25,25.23)	34.45 (24.53,49.48)	-7.022	0.002
FPG (mmol/L)	5.48 (5.12, 5.80)	5.07 (4.89,5.31)	-4.975	0.002
FINS (μU/mL)	24.35 (17.13,34.03)	8.60 (6.38,9.98)	-11.363	0.002
HOMA-IR	5.86 (4.05,8.35)	2.05 (1.44,2.26)	-11.483	<0.001
HOMA-β	245.42 (174.74, 377.62)	104.01 (75.42,121.43)	-9.518	0.002
TG (mmol/L)	1.49 (1.03, 1.99)	0.93 (0.75,1.31)	-5.158	0.002
FT3 (pmol/L)	4.70 (4.31,5.08)	4.46 (4.18,4.76)	-2.607	0.015
FT4 (pmol/L)	12.74 (11.71,14.03)	13.06 (11.94, 14.25)	-1.039	0.313
FT3/FT4	0.36 (0.34,0.40)	0.34 (0.30,0.39)	-2.439	0.022
TSH (mIU/L)	1.95 (1.47,2.78)	1.45 (1.15,2.32)	-3.193	0.002
TSHI	2.40 (2.02,2.83)	2.19 (1.90, 2.61)	-1.986	0.064
TT4RI	25.25 (18.06,37.38)	19.39 (15.21, 30.15)	-2.444	0.022

Normally distributed data are presented as mean ± SD; non-normally distributed data are presented as median (Q1, Q3);HOMA-IR was the sole primary outcome of this study, and its P values were unadjusted. P-values for other secondary exploratory variables were adjusted for multiple comparisons using the Benjamini–Hochberg FDR method. SBP, systolic blood pressure; DBP, diastolic blood pressure; BMI, body mass index; WC, waist circumference; mFG score, modified Ferriman-Gallwey score; FSH, follicle-stimulating hormone; LH, luteinizing hormone; T, testosterone; DHEAS, dehydroepiandrosterone sulfate; SHBG, sex hormone-binding globulin; FPG, fasting plasma glucose; FINS, fasting insulin; HOMA-IR, homeostasis model assessment of insulin resistance; HOMA-β, homeostasis model assessment of β-cell function; TG, triglycerides; FT3, free triiodothyronine; FT4, free thyroxine; TSH, thyroid- stimulating hormone; TSHI, thyroid-stimulating hormone index; TT4RI, thyrotroph thyroxine resistance index; SD, standard deviation.

**Table 2:** Comparison of clinical indicators by TT4RI levels in phlegm-dampness syndrome PCOS patients.

Variable	Group			*F(H)*	*P*
	LTT4RI (n=88)	MTT4RI (n=88)	HTT4RI (n=88)		
Weight (kg)	75.00 (65.00,83.25)	77.50 (70.00,82.88)	80.00 (72.13, 90.00)^c^	6.639	0.077
BMI(kg/m^2^)	28.06 (25.23,31.16)	28.69 (26.27,30.95)	30.19 (25.80,32.07)	5.438	0.104
WC (cm)	91.74 ± 13.33	92.70 ± 11.51	95.14 ± 11.74	1.808	0.203
FPG(mmol/L)	5.24 (4.94,5.57)	5.47 (5.05,5.74)	5.43 (5.10,5.77)	5.081	0.109
FINS(μU/mL)	16.45 (10.78, 29.35)	18.85 (12.55, 29.20)	25.55 (16.25,34.00)^c^	9.093	0.030
HOMA-IR	3.85 (2.45, 6.89)	4.79 (2.92,7.07)	6.00 (3.99,8.75)^c^	8.746	0.013
HOMA-β	192.79 (120.16,324.45)	194.98 (121.62,276.36)	251.05 (164.15,366.07)	6.348	0.077
TG(mmol/L)	1.40 (0.88,1.94)	1.35 (0.89,1.92)	1.36 (0.99,1.87)	0.496	0.780
FT3(pmol/L)	4.60 (4.28,4.99)	4.56 (4.25,5.03)	4.67 (4.35,5.10)	1.450	0.532
FT4(pmol/L)	12.23 (11.33,13.26)	12.74 (11.84, 13.77)	13.45 (12.19,15.48)^bc^	22.867	0.004
FT3/FT4	0.37 (0.34,0.41)	0.36 (0.34,0.39)	0.35 (0.31,0.38)	13.245	0.004
TSH(mIU/L)	1.16 (1.00,1.39)	1.86 (1.70,2.24)^a^	3.09 (2.63,4.02)^bc^	210.894	0.004

Normally-distributed: ANOVA-LSD; non-normally-distributed: Kruskal-Wallis with Dunn-Bonferroni post-hoc comparisons. HOMA-IR was the sole primary outcome of this study, and its P values were unadjusted. P-values for other secondary exploratory variables were adjusted for multiple comparisons using the Benjamini–Hochberg FDR method.; ^a^Significant difference between LTT4RI and MTT4RI; ^b^Significant difference between MTT4RI and HTT4RI; ^c^Significant difference between LTT4RI and HTT4RI. LTT4RI,the low TT4RI tertile group (n=88);MTT4RI,the middle TT4RI tertile group (n=88); HTT4RI, the high TT4RI tertile group (n=88); BMI, body mass index; WC, waist circumference; FPG, fasting plasma glucose; FINS, fasting insulin; HOMA-IR, homeostasis model assessment of insulin resistance; HOMA-β, homeostasis model assessment of β-cell function; TG, triglycerides; FT3, free triiodothyronine; FT4, free thyroxine; TSH, thyroid- stimulating hormone.

**Table 3:** Correlation analysis.

Correlations	*r*	*P*
Weight (kg)	0.162*	0.018
BMI(kg/m^2^)	0.140*	0.040
WC (cm)	0.119	0.073
FPG(mmol/L)	0.135*	0.046
FINS(μU/mL)	0.167**	0.018
HOMA-IR	0.169**	0.006
HOMA-β	0.115	0.076
TG(mmol/L)	0.024	0.694
FT3(pmol/L)	0.060	0.363
FT4(pmol/L)	0.333**	0.003
FT3/FT4	-0.261**	0.003
TSH(mIU/L)	0.943**	0.003

HOMA-IR was the sole primary outcome of this study, and its P values were unadjusted. P-values for other secondary exploratory variables were adjusted for multiple comparisons using the Benjamini–Hochberg FDR method; *P < 0.05; **P < 0.01; ***P < 0.001;BMI,body mass index; WC, waist circumference; FPG, fasting plasma glucose; FINS, fasting insulin; HOMA-IR, homeostasis model assessment of insulin resistance; HOMA-β, homeostasis model assessment of β-cell function; TG, triglycerides; FT3, free triiodothyronine; FT4, free thyroxine; TSH, thyroid- stimulating hormone.

Natural log-transformed lnHOMA-IR was adopted for multivariable linear regression modeling to meet the statistical assumption of residual normality. The P-values from multivariable regression models are reported as raw P-values, and regression analyses were not included in the multiple-comparison correction for baseline characteristics. Furthermore, we performed a sensitivity analysis by constructing multiple linear regression models with Z-standardized TT4RI as the continuous independent variable and lnHOMA-IR as the dependent variable. We verified the assumptions of multivariable linear regression, including linearity between independent variables and outcome, normality of residuals, homoscedasticity, and no severe multicollinearity (variance inflation factor, VIF < 5). Covariates including age, BMI, SBP, and SHBG were selected *a priori* based on clinical pathophysiology and previous studies. These variables are known confounders associated with both thyroid function markers and insulin resistance (13).

Receiver-operating characteristic (ROC) curve analysis was applied to compare the diagnostic performance of TT4RI, TSHI, and FT3/FT4 ratio for identifying insulin resistance in patients with phlegm-dampness syndrome PCOS.

## 3 Results

HOMA-IR was the sole primary outcome with unadjusted P-values. Other clinical indices in

**Tables 1**–**3** were defined as secondary exploratory variables, and their P-values were FDR-corrected. Multivariable regression used lnHOMA-IR (log-transformed HOMA-IR) as the dependent variable, reporting raw unadjusted P-values.

### 3.1 Baseline characteristics

**Table 4** shows the distribution of insulin resistance among PCOS patients with and without phlegm-dampness syndrome. A total of 486 PCOS patients were included in the study: 264 (54.32%) with phlegm-dampness syndrome and 222 (45.68%) without phlegm-dampness syndrome. Among the patients with phlegm-dampness syndrome, 208 had insulin resistance, while 56 did not. This difference was statistically significant (P < 0.05).

**Table 4:** Distribution of insulin resistance in PCOS patients with and without phlegm-dampness syndrome.

Group	*n*	IR(%)	Non-IR(%)	χ²	*P*
Phlegm-dampness syndrome	264 (54.32%)	208 (42.80%)	56 (11.52%)	11.706	<0.001
Non-phlegm-dampness syndrome	222 (45.68%)	144 (29.63%)	78 (16.05%)		

IR, PCOS with insulin resistance; Non-IR, PCOS without insulin resistance. Insulin resistance was defined as HOMA-IR > 2.65.

**Table 1** shows the baseline characteristics of phlegm-dampness syndrome PCOS patients with and without IR. Among patients with phlegm-dampness syndrome PCOS, those with IR had significantly higher levels of multiple clinical and biochemical indicators compared to those without IR, including: systolic and diastolic blood pressure, anthropometric measures (such as body weight, BMI and waist circumference), modified Ferriman-Gallwey score and acanthosis nigricans score, glucose metabolism indicators (such as FPG, FINS, HOMA-IR and HOMA-β), TG and thyroid-related indicators (e.g.FT_3_, FT_3_/FT_4_, TSH, and TT4RI). All differences were statistically significant (P < 0.05). In the PCOS with phlegm-dampness syndrome group accompanied by IR, SHBG was lower than in the group without IR (P < 0.05). Differences in other clinical indicators were not statistically significant (P > 0.05).

### 3.2 Comparison of clinical indicators across different TT4RI levels in phlegm-dampness syndrome PCOS patients

PCOS patients with phlegm-dampness syndrome were grouped into three tertiles based on their TT4RI levels from low to high: the low TT4RI tertile (LTT4RI) group (TT4RI ≤ 18.98, n = 88), the middle TT4RI tertile (MTT4RI) group (18.98 < TT4RI ≤ 32.46, n = 88), and the high TT4RI tertile (HTT4RI) group (TT4RI > 32.46, n = 88). The differences in clinical indicators among the three groups were compared. There were statistically significant differences (P < 0.05) among the three groups in FINS, HOMA-IR, FT4, FT3/FT4, and TSH; no significant differences were observed for other indicators (P > 0.05). For FT4 and TSH, the measured values in the HTT4RI group were higher than those in the LTT4RI and MTT4RI groups, and the differences between groups were statistically significant (P < 0.05).In the HTT4RI group, body weight, HOMA-IR, and FINS were all higher than in the LTT4RI group. In the MTT4RI group, TSH was higher than in the LTT4RI group, with statistically significant differences (P < 0.05). (see **Table 2**).

### 3.3 Correlation analyses

Spearman’s correlation analyses were used to investigate the correlation between the TT4RI and various clinical indicators in patients with phlegm-dampness syndrome PCOS. The results showed that TT4RI was positively correlated with anthropometric indicators (including body weight and BMI), FPG, FINS, HOMA-IR, FT4, and TSH (r > 0, P < 0.05), and was negatively correlated with the FT3/FT4 ratio (r < 0, P < 0.05). (see **Table 3**).

### 3.4 Multivariable linear regression analyses

In **Table 5**, stratified multivariable linear regression analyses were applied to explore the association between TT4RI and IR. Patients with PCOS presenting with phlegm-dampness syndrome were stratified by TT4RI into three tertiles: the LTT4RI group (serving as the reference group), the MTT4RI group, and the HTT4RI group. A stratified multiple linear regression model was constructed using the lnHOMA-IR as the outcome variable. In the crude model (Model 1), with the LTT4RI group as the reference group, the HTT4RI group showed a relatively higher level of lnHOMA-IR (β= 0.275, 95% CI: 0.059-0.490, *P* = 0.013), and the trend test indicated a positive correlation between TT4RI and lnHOMA-IR (*P* = 0.013); After adjusting for age and BMI (Model 2), the association between the HTT4RI group and lnHOMA-IR was not statistically significant (β= 0.144, 95% CI: −0.048-0.335, *P* = 0.141), and the trend test was also not statistically significant (*P* = 0.141); Upon further adjustment for SBP and SHBG (Model 3), the HTT4RI group showed marginal statistical significance compared with the LTT4RI group (β= 0.177, 95% CI: 0.002-0.352, *P* = 0.048), and the trend test was also marginally positive (*P* = 0.048); In all three models, the MTT4RI group showed no statistical significance compared with the reference group (P > 0.05).

**Table 5:** Stratified linear regression of TT4RI and IR in phlegm-dampness syndrome PCOS patients.

Model1			Model2			Model3		
Tertile groups	β (95%CI)	*P*	Tertile groups	β (95%CI)	*P*	Tertile groups	β (95%CI)	*P*
LTT4RI (n=88)	1.00 (Reference)		LTT4RI	1.00 (Reference)		LTT4RI	1.00 (Reference)	
MTT4RI (n=88)	0.042 (-0.174,0.257)	0.705	MTT4RI	-0.042 (-0.233,0.148)	0.662	MTT4RI	-0.021 (-0.196,0.153)	0.810
HTT4RI (n=88)	0.275 (0.059,0.490)	0.013	HTT4RI	0.144 (-0.048,0.335)	0.141	HTT4RI	0.177 (0.002,0.352)	0.048
*P* value for trend		0.013	*P* value for trend		0.141	*P* value for trend		0.048

Multiple Linear Regression Models: Model 1 is the crude model; Model 2 adjusts for age and BMI; Model 3 adjusts for age, BMI, SBP, and SHBG; β, unstandardized regression coefficient; 95% CI, 95% confidence interval.

LTT4RI, the low TT4RI tertile group (n=88); MTT4RI, the middle TT4RI tertile group (n=88); HTT4RI, the high TT4RI tertile group (n=88).

Additionally, Sensitivity analysis further characterized the association between TT4RI and insulin resistance, in which Z-standardized TT4RI was treated as a continuous variable and incorporated into multivariable linear regression models with lnHOMA-IR as the dependent variable (**Supplementary Table S2**). In model 1, TT4RI showed a marginal trend toward positive association with lnHOMA-IR (β=0.079, 95%CI: -0.010 to 0.167, *P* = 0.081). After adjustment for age and BMI, this association attenuated and became non-significant(β=0.040, 95%CI: -0.038 to 0.118, *P* = 0.313. Further adjustment for age, BMI, SBP, and SHBG still revealed no independent linear association between TT4RI and lnHOMA-IR (β=0.038, 95%CI: -0.034 to 0.110, *P* = 0.299).

### 3.5 Diagnostic performance of thyroid sensitivity indices for IR in phlegm-dampness syndrome PCOS patients

This study employed ROC curve analysis, and the results suggest that TT4RI, TSHI, and the FT3/FT4 ratio all have the potential to identify insulin resistance in PCOS patients with phlegm-dampness syndrome, with AUC values of 0.606, 0.586, and 0.606, respectively (P < 0.05). Based on Youden’s index, the optimal cutoff value for TT4RI was determined to be 18.84, at which sensitivity reached 72.1%. While its screening sensitivity was superior to that of TSHI and the FT3/FT4 ratio, its specificity was limited. Overall, ROC analysis shows that TT4RI demonstrates limited discriminatory performance in distinguishing insulin resistance in PCOS patients with phlegm-dampness syndrome. (See **Table 6**; **Figure 2**).

**Table 6:** Diagnostic performance of thyroid sensitivity indices for IR in phlegm-dampness syndrome PCOS patients.

Biomarker	AUC	95% CI lower	95% CI upper	Optimal cut-off	Sensitivity	Specificity	*P*
TT4RI	0.606	0.521	0.691	18.84	72.10%	50.00%	0.015
TSHI	0.586	0.500	0.673	2.12	70.20%	48.20%	0.047
FT3/FT4	0.606	0.515	0.697	0.35	64.90%	60.70%	0.015

Optimal cut-off values were obtained by maximizing Youden’s index. These thresholds are exploratory and derived from this single-center retrospective observational analysis. TSHI, thyroid-stimulating hormone index; TT4RI, thyrotroph thyroxine resistance index.

**Figure 2:**
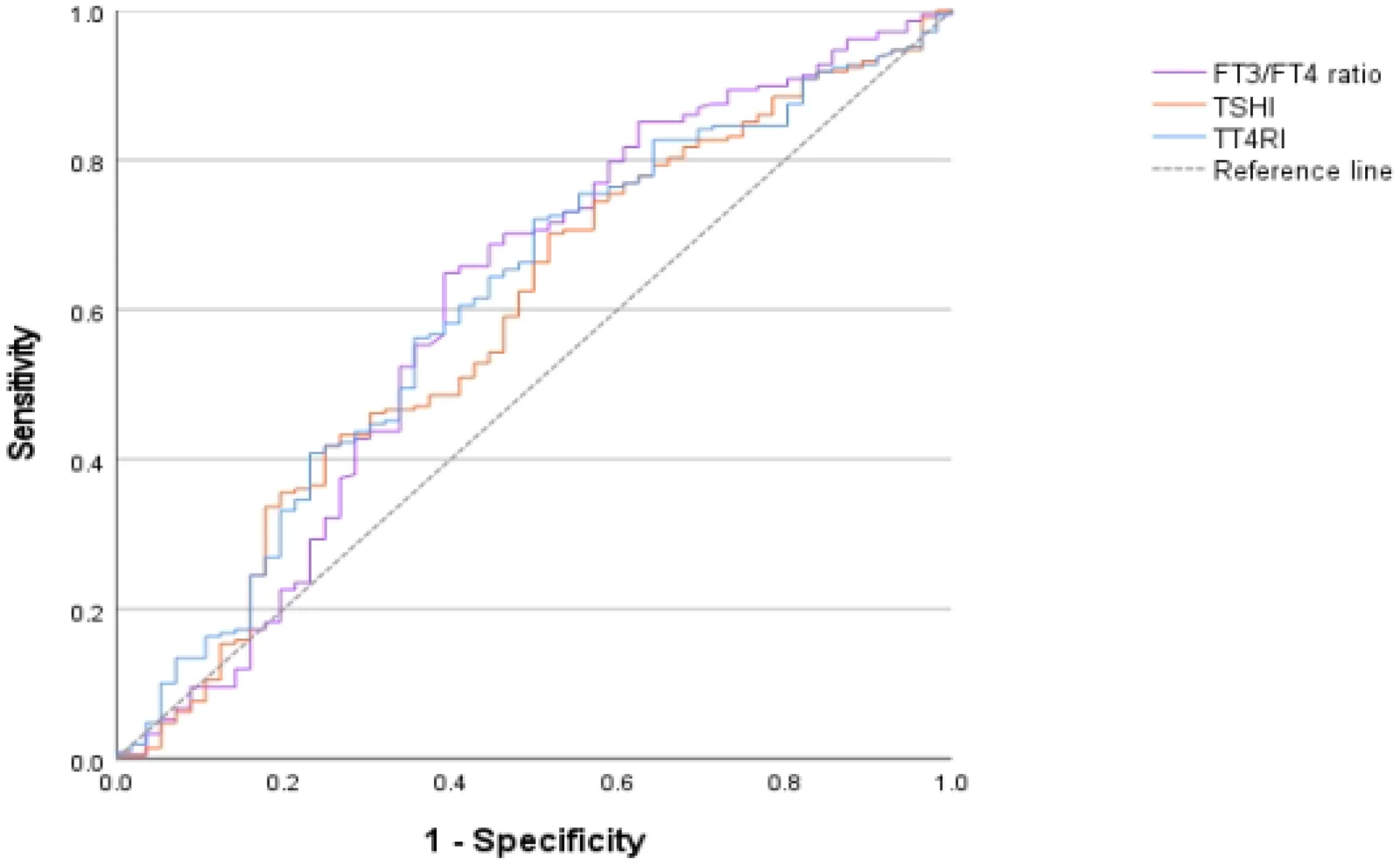
ROC curves of TT4RI, TSHI and FT3/FT4 ratio for detecting insulin resistance in patients with phlegm-dampness syndrome PCOS.X-axis, 1-Specificity; Y-axis, Sensitivity.

## 4 Discussion

As a common reproductive endocrine disorder in women, PCOS is associated with genetic factors, metabolic disorders, and dysregulation of the hypothalamic-pituitary-ovarian axis, and is often accompanied by insulin resistance, type 2 diabetes, and thyroid dysfunction. In recent years, it has been discovered that thyroid hormones, in addition to playing a broad role in endocrine and metabolic regulation, are also key regulators in maintaining female reproductive health. Insulin resistance is the core pathological mechanism mediating glucose and lipid metabolism disorders in PCOS with phlegm-dampness syndrome, while thyroid dysfunction can interfere with insulin signaling through the hypothalamic-pituitary-thyroid axis and inflammatory pathways. These often interact to jointly cause metabolic abnormalities in PCOS. However, previous clinical studies have primarily focused on single thyroid markers such as FT3, FT4, and TSH, and no studies have yet reported on the central thyroid hormone sensitivity index in patients with phlegm-dampness syndrome PCOS. This study conducted a retrospective observational analysis based on this approach to clarify the intrinsic association between TT4RI and insulin resistance in phlegm-dampness syndrome PCOS. This study was retrospective, and only the final adjudicated syndrome diagnosis was recorded in the electronic medical system. Therefore, inter-rater reliability analysis (Cohen’s Kappa) could not be performed. Nevertheless, strict diagnostic procedures, including standardized training, independent double evaluation, and senior expert arbitration, ensured the reliability of phlegm-dampness syndrome differentiation.

This study included a total of 264 patients with PCOS characterized by phlegm-dampness syndrome, of whom 208 had insulin resistance. In the group with PCOS characterized by phlegm-dampness syndrome and IR, SBP, DBP, modified Ferriman-Gallwey score, and acanthosis nigricans score were all higher than in the group without IR (P < 0.05). In addition, glucose and lipid metabolism indicators, such as FPG, FINS, HOMA-IR, HOMA-β, and TG, were all higher in the group with IR than in the group without IR (P < 0.05). It should be noted that patients in this study were classified into IR and Non-IR groups based on the HOMA-IR cutoff value. Since HOMA-IR is derived from FPG and FINS, inter-group differences in HOMA-IR, FINS, and FPG are inherent to this grouping definition and do not constitute independent novel findings of the present study. The core of this stratified analysis is to compare thyroid-sensitivity indices such as TT4RI between the two groups, laying a foundation for further exploring the association between thyroid hormone sensitivity and insulin resistance in patients with phlegm-dampness syndrome PCOS.IR refers to a diminished ability of insulin to promote glucose uptake by peripheral tissues, accompanied by impaired inhibition of hepatic glucose output. In patients with PCOS, prolonged insulin resistance gradually exacerbates the severity of their glucose and lipid metabolism disorders. Consequently, patients with PCOS characterized by phlegm-dampness syndrome often exhibit abdominal obesity, with significantly elevated body weight, waist circumference, and BMI levels. The degree of obesity in patients is negatively correlated with insulin sensitivity; excessive obesity can lead to increased secretion by pancreatic β-cells, resulting in impaired β-cell function and subsequent diabetes. A study by Pan Zimeng et al. (3) showed that, compared with PCOS patients without the phlegm-dampness syndrome, those with the phlegm-dampness syndrome exhibited a more pronounced compensatory hyperinsulinemic response under both fasting and glucose-loaded conditions. As stated in Danxi’s Heart Method: “Obese people often have phlegm-dampness.” Patients’ craving for rich, sweet, and greasy foods excessively generates phlegm-dampness; when dampness accumulates over time in the middle jiao (spleen and stomach) or overflows to the skin, it can lead to obesity. Furthermore, due to hormonal imbalances, PCOS patients often develop light brown or black skin pigmentation on the neck, armpits, breasts, and thighs, a condition known as acanthosis nigricans. This is also a cutaneous marker for insulin resistance (14). This study further confirms that PCOS patients with phlegm-dampness syndrome and insulin resistance have significantly higher acanthosis nigricans scores than those without IR. Lipid metabolism abnormalities are commonly observed in women with PCOS; significant differences in various components, including triglycerides, glycerophospholipids, sphingolipids, and carboxylic acids, are present in their follicular fluid (15), and insulin resistance may be indirectly caused by lipotoxicity (16).

Studies have found that thyroid receptors and thyroid-stimulating hormone receptors are expressed in both the ovaries and the uterus (17). Abnormal thyroid function can directly interfere with normal gonadal function and fertility, leading to delayed puberty and anovulatory cycles, and thus serves as one of the potential driving factors in the onset and progression of PCOS in women. TT4RI and TSHI, as indicators of central sensitivity, reflect the state of thyroid hormone resistance in the pituitary and hypothalamus. Higher TSHI and TT4RI values indicate lower central sensitivity and thyroid hormone resistance. Thyroid hormone resistance in the general population is often associated with a high prevalence of metabolic diseases such as hypertension and diabetes (18). This study also confirmed that in patients with phlegm-dampness syndrome PCOS, those with IR had higher FT3, FT3/FT4, TSH, and TT4RI indices compared to those without IR. Furthermore, in the HTT4RI group, HOMA-IR and FINS were both significantly higher than in the LTT4RI group. This indirectly indicates that thyroid hormone sensitivity is associated with the severity of insulin resistance in PCOS patients with phlegm-dampness syndrome. Wang Q etal. (19), in a study involving 415 PCOS patients, found an association between reduced central thyroid hormone sensitivity and an increased risk of PCOS. This study shows that TSH levels increase as TT4RI levels rise. Higher TSH levels indicate hypothyroidism, which in clinical practice often leads to metabolic disorders in PCOS. Research has shown (20) that TSH may exacerbate inflammation by promoting macrophage infiltration and activating the TLR4 inflammatory pathway, thereby disrupting hepatic insulin signaling. The results showed that in the group of rats with subclinical hypothyroidism, levels of TLR4 and downstream molecules(such as tumor necrosis factor-α, phosphorylated NF-κB, phosphorylated IRAK-1, and interleukin-6)were significantly higher than in the normal control group. Furthermore, the group of rats with subclinical hypothyroidism exhibited lower glucose uptake rates and higher levels of free fatty acids, FINS, and HOMA-IR. Shekarian et al. (21) conducted a meta-analysis of 5,765 women with PCOS, which showed that 19.7% of these women had subclinical hypothyroidism. Compared to PCOS patients without subclinical hypothyroidism, those with the condition had significantly higher HOMA-IR and fasting insulin levels. At the same time, a case-control study (22) confirmed that among women with PCOS, those with concomitant autoimmune thyroid disease (AITD) had significantly higher rates of obesity and higher HOMA-IR levels than those without AITD.

The above results suggest that autoimmune thyroid disease may be associated with an adverse metabolic phenotype in women with PCOS. Martinez et al. (23) suggest that thyroid dysfunction may exacerbate insulin resistance in women with PCOS by altering insulin receptor sensitivity. Animal studies have shown (24) that triiodothyronine can inhibit the excessive activation of glycogen synthase kinase 3 by specifically restoring insulin signaling pathways in the hippocampus of diabetic rats, thereby reducing the abnormal accumulation of tau protein and ultimately lowering blood glucose levels and alleviating pathological changes in the brain. Thyroid hormones regulate glucose and lipid metabolism. The prevalence of thyroid disorders, particularly subclinical abnormalities, is significantly elevated in women with PCOS (25).

This study found that the TT4RI showed positive correlations with body weight, BMI, FPG, FINS, HOMA-IR, FT4, and TSH indices in PCOS patients with the phlegm-dampness syndrome (r > 0, P < 0.05). It should be noted that TT4RI is a derived index calculated from TSH and FT4. Therefore, their high correlation partly arises from inherent mathematical associations caused by shared calculation parameters and cannot be fully interpreted as an independent physiological relationship. We may place more focus on the association between TT4RI and insulin resistance. As an indicator of peripheral thyroid sensitivity, the FT3/FT4 ratio reflects the ability of peripheral tissues to convert FT4 into FT3. A higher ratio indicates greater peripheral thyroid sensitivity (18).

In this study, among PCOS patients with phlegm-dampness syndrome, the crude association between TT4RI and insulin resistance was significantly weakened after adjusting for metabolic confounders such as BMI. SHBG synthesis is regulated by insulin and sex hormones, and SBP is a component of metabolic syndrome. Both are associated with the independent variable TT4RI and the outcome variable lnHOMA-IR. After further adjustment for SBP and SHBG, only the HTT4RI group showed marginal statistical significance in its association with IR. Sensitivity analysis further characterized the association between TT4RI and insulin resistance. No linear dose-response relationship was observed across the full range of TT4RI values. These findings suggest that TT4RI may exert a threshold effect on insulin resistance: the association becomes apparent only when TT4RI rises above a certain level. According to our ROC-curve analysis, the optimal TT4RI cut-off value was 18.84 in the present study, implying that TT4RI above this threshold may be associated with an elevated association with insulin resistance. Nevertheless, TT4RI shows limited discriminatory performance in identifying IR among patients with phlegm-dampness syndrome PCOS. The cut-off value obtained in this study was derived from a single-center retrospective analysis, which should be regarded as exploratory and requires independent prospective validation before any clinical screening implications can be considered. Furthermore, the relationship between TT4RI and insulin resistance is susceptible to interference from metabolic covariates. Therefore, the independent predictive value of TT4RI for insulin resistance is limited due to the limited sample size of this study and the fact that statistical power is insufficient when indirect effects are weak, which can easily lead to false results; mediation analysis has not yet been conducted. Future studies could expand the sample size to further investigate whether metabolic covariates mediate the association between TT4RI and insulin resistance in women with PCOS presenting with phlegm-dampness syndrome. Additionally, this study had significant missing data regarding patients’ smoking history, and the use of oral contraceptives was not systematically recorded, which may introduce residual confounding bias. Although TT4RI has not yet demonstrated significant added value, this study provides baseline reference data on TT4RI in the phlegm-dampness syndrome of PCOS, which can serve as a basis for future studies on the subtyping of thyroid sensitivity markers and insulin resistance.

## 5 Conclusion

In summary, this study suggests that reduced central thyroid hormone sensitivity is associated with an increased association of insulin resistance in women with PCOS presenting with phlegm-dampness syndrome. Simple correlation analysis revealed that the TT4RI showed weak positive correlations with body weight, BMI, FPG, FINS, HOMA-IR, FT4 and TSH indices in PCOS patients with the phlegm-dampness syndrome, and negatively correlated with the FT3/FT4 ratio. Multivariate linear regression analysis revealed a certain association between TT4RI and lnHOMA-IR. However, after adjusting for confounding factors such as age, BMI, SBP, and SHBG, the independent association between TT4RI and lnHOMA-IR weakened. These findings provide a basis for future prospective studies. The potential molecular mechanisms underlying this association remain unclear. This study has several limitations. First, it is a single-center retrospective observational analysis with a relatively modest sample size. Second, no intervention trials have yet been conducted to verify the association between TT4RI and insulin resistance. In the future, it will be necessary to conduct multicenter, prospective cohort studies to further investigate the relationship between the thyroid-related biomarker TT4RI and HOMA-IR, elucidate their potential molecular mechanisms, and explore its potential clinical value.

## Data Availability

All raw clinical data supporting the findings of this study are derived from the electronic medical record system of The First Affiliated Hospital of Heilongjiang University of Chinese Medicine. The datasets contain de-identified clinical indicators of patients with phlegm-dampness and polycystic ovary syndrome collected from February 2023 to September 2025. Restrictions apply to the availability of these data, as the original medical records involve patients’ private clinical information and cannot be publicly shared to protect patient confidentiality. Requests to access the datasets should be directed to the corresponding author with reasonable ethical approval. Requests to access these datasets should be directed to Hongying Kuang, E-mail: hyk20042@sina.com.

## References

[1] Di Lorenzo M Cacciapuoti N Lonardo MS Nasti G Gautiero C Belfiore A . Pathophysiology and nutritional approaches in polycystic ovary syndrome (PCOS): a comprehensive review. Curr Nutr Rep. (2023) 12:527–44. doi: 10.1007/s13668-023-00479-8 37213054 PMC10444658

[2] Zhao H Zhang J Cheng X Nie X He B . Insulin resistance in polycystic ovary syndrome across various tissues: an updated review of pathogenesis, evaluation, and treatment. J Ovarian Res. (2023) 16:9. doi: 10.1186/s13048-022-01091-0 36631836 PMC9832677

[3] Pan ZM Li J Sun C Han PG Sun M Xu F . Correlation analysis of insulin sensitivity, isletβ-cell function and clinical characteristics in polycystic ovary syndrome patients with phlegm-dampness syndrome. China J Chin Med. (2022) 37:5950–6. In Chinese.

[4] Palomba S Colombo C Busnelli A Caserta D Vitale G . Polycystic ovary syndrome and thyroid disorder: a comprehensive narrative review of the literature. Front Endocrinol (Lausanne). (2023) 14:1251866. doi: 10.3389/fendo.2023.1251866 37635968 PMC10453810

[5] Cui R Li G Wei Y Liu J Wang Y Leng S . Thyroid hormone resistance indices and their correlations with insulin resistance in Chinese euthyroid subjects. Int J Endocrinol. (2025) 2025:5397686. doi: 10.1155/ije/5397686 41040505 PMC12488306

[6] Laclaustra M Moreno-Franco B Lou-Bonafonte JM Mateo-Gallego R Casasnovas JA Guallar-Castillon P . Impaired sensitivity to thyroid hormones is associated with diabetes and metabolic syndrome. Diabetes Care. (2019) 42:303–10. doi: 10.2337/dc18-1410 30552134

[7] Nie X Ma X Xu Y Shen Y Wang Y Bao Y . Increased serum adipocyte fatty acid-binding protein levels are associated with decreased sensitivity to *thyroid* hormones in the eu*thyroid* population. Thyroid. (2020) 30:1718–23. doi: 10.1089/thy.2020.0011 32394790

[8] Kachuei M Jafari F Kachuei A Keshteli AH . Prevalence of autoimmune thyroiditis in patients with polycystic ovary syndrome. Arch Gynecol Obstet. (2012) 285:853–6. doi: 10.1007/s00404-011-2040-5 21866332

[9] Glintborg D Rubin KH Nybo M Abrahamsen B Andersen M . Increased risk of thyroid disease in Danish women with polycystic ovary syndrome: a cohort study. Endocr Connect. (2019) 8:1405–15. doi: 10.1530/EC-19-0377 31518989 PMC6826171

[10] Endocrinology Group, Obstetrics and Gynecology Branch of Chinese Medical Association Guideline Expert Panel . Chinese guidelines for the diagnosis and treatment of polycystic ovary syndrome. Chin J Obstetrics Gynecology. (2018) 53:2–6. doi: 10.3760/cma.j.issn.0529-567x.2018.01.002 29374878

[11] Department of Medical Administration, National Administration of Traditional Chinese Medicine . Clinical Pathways of Traditional Chinese Medicine for 105 Diseases in 24 Specialties (Trial). Beijing: National Administration of Traditional Chinese Medicine (2011) p. 532–3.

[12] Zhou L Lv F Xu MQ Dong DT Ni J . Correlation between insulin resistance and clinical pregnancy rate of intrauterine insemination with husband semen in infertile patients with polycystic ovary syndrome. Chin J Clin Obstetrics Gynecology. (2025) 26:437–40. doi: 10.13390/j.issn.1672-1861.2025.05.015

[13] Ghobrial S Krysiak R Goldstein T Patsch A Paternostro C Heinzl F . Distribution of prolactin and its correlation with insulin resistance in women with polycystic ovary syndrome. Front Endocrinol (Lausanne). (2025) 16:1674795. doi: 10.3389/fendo.2025.1674795 41103658 PMC12520901

[14] Rafique S Dihowm H Parveen S Jakoby M . Insulin-induced acanthosis nigricans. Cureus. (2025) 17:e83424. doi: 10.7759/cureus.83424 40458329 PMC12129569

[15] Liu L Yin TL Chen Y Li Y Yin L Ding J . Follicular dynamics of glycerophospholipid and sphingolipid metabolisms in polycystic ovary syndrome patients. J Steroid Biochem Mol Biol. (2019) 185:142–9. doi: 10.1016/j.jsbmb.2018.08.008 30121347

[16] Yazıcı D Demir SÇ Sezer H . Insulin resistance, obesity, and lipotoxicity. Adv Exp Med Biol. (2024) 1460:391–430. doi: 10.1007/978-3-031-63657-8_14 39287860

[17] Jia C Zhang L Liu W Zhang X Wu H . Assessment of glucose and lipid metabolism in patients with polycystic ovary syndrome with and without Hashimoto's thyroiditis. Med (Baltimore). (2023) 102:e33205. doi: 10.1097/MD.0000000000033205 36930082 PMC10019210

[18] Sun WW Niu YH . Relationship between thyroid hormone resistance and coronary atherosclerosis. Lingnan J Cardiovasc Dis. (2025) 31:40–4. doi: 10.3969/j.issn.1007-9688.2025.01.07

[19] Wang Q Zhao R Han C Huang Z Bi Y Zhang X . Correlation between thyroid hormone sensitivity and the risk of polycystic ovary syndrome. BMC Endocr Disord. (2024) 24:76. doi: 10.1186/s12902-024-01607-3 38816692 PMC11137887

[20] Bao S Li F Duan L Li J Jiang X . Thyroid-stimulating hormone may participate in insulin resistance by activating toll-like receptor 4 in liver tissues of subclinical hypothyroid rats. Mol Biol Rep. (2023) 50:10637–50. doi: 10.1007/s11033-023-08834-2 37884783

[21] Shekarian A Mazaheri-Tehrani S Shekarian S Pourbazargan M Setudeh M Abhari AP . Prevalence of subclinical hypothyroidism in polycystic ovary syndrome and its impact on insulin resistance: a systematic review and meta-analysis. BMC Endocr Disord. (2025) 25:75. doi: 10.1186/s12902-025-01896-2 40102852 PMC11921581

[22] Kim JJ Yoon JW Kim MJ Kim SM Hwang KR Choi YM . Thyroid autoimmunity markers in women with polycystic ovary syndrome and controls. Hum Fertil (Camb). (2022) 25:128–34. doi: 10.1080/14647273.2019.1709668 31910041

[23] Martinez B Ortiz RM . Thyroid hormone regulation and insulin resistance: insights from animals naturally adapted to fasting. Physiol (Bethesda). (2017) 32:141–51. doi: 10.1152/physiol.00018.2016 28202624

[24] Prieto-Almeida F Panveloski-Costa AC Crunfli F da Silva Teixeira S Nunes MT Torrão ADS . Thyroid hormone improves insulin signaling and reduces the activation of neurodegenerative pathway in the hippocampus of diabetic adult male rats. Life Sci. (2018) 192:253–8. doi: 10.1016/j.lfs.2017.11.013 29132997

[25] You Y Li X Guo Z Hong X Yu Q . Effect of impaired sensitivity to thyroid hormones on the risk of insulin resistance and dyslipidemia in polycystic ovary syndrome patients. Gynecol Endocrinol. (2025) 41:2537275. doi: 10.1080/09513590.2025.2537275 41025423

