# Supplementary material for: Correlation between the thyrotropin-thyroxine resistance index and insulin resistance in polycystic ovary syndrome patients with phlegm-dampness syndrome
Source: Front Endocrinol (Lausanne). 2026 Oct 8;17:1940904. doi: 10.3389/fendo.2026.1940904 (PMC13650464; doi:10.3389/fendo.2026.1940904)
Supplement: Supplementary file 1 [file Table1.docx]

**Table S1.** Assay performance and laboratory reference ranges for serological indicators

| Serological indicator | Intra‑assay CV (%) | Inter‑assay CV (%) | Laboratoryreference range |
| --- | --- | --- | --- |
| FSH(mIU/mL） | 5.82 | 5.88 | Males: 0.95‑11.95; Females: follicular phase: 3.03‑8.08; mid‑cycle: 2.55‑16.69; luteal phase: 1.38‑5.47; postmenopausal females: 26.72‑133.41. |
| LH(mIU/mL） | 5.03 | 5.12 | Males:0.57‑12.07;Females:follicular phase:1.80‑11.78;mid‑cycle peak: 7.59‑89.08; luteal phase: 0.56‑14.00; postmenopausal females (without HRT): 5.16‑61.99. |
| T(nmol/L) | 6.84 | 6.88 | Males:4.94-32.01；Females: 0.38-1.97 |
| DHEAS（μmol/L） | Not archived | Not archived | 1.53-13.89 |
| SHBG（nmol/L） | Not archived | Not archived | 19.80-155.20 |
| FPG(mmol/L） | 1.26 | 1.31 | 3.89-6.11 |
| FINS(μU/mL） | 2.94 | 2.99 | 2.30-11.80 |
| TG（mmol/L） | 1.08 | 1.21 | 0.56-1.70 |
| FT3(pmol/L） | 6.21 | 6.26 | 2.43-6.02 |
| FT4(pmol/L） | 6.26 | 6.31 | 9.01-19.05 |
| TSH(mIU/L） | 3.63 | 3.66 | 0.35-4.94 |

CV: coefficient of variation. “Not archived” indicates that intra‑assay and inter‑assay CV records were not retained in laboratory archives. All CV values were derived from medium‑level quality‑control samples of routine laboratory internal quality‑control records.FSH,follicle-stimulating hormone；LH,luteinizing hormone；T,testosterone;DHEAS,dehydroepiandrosterone sulfate;SHBG,sex hormone-binding globulin;FPG,fasting plasma glucose;FINS,fasting insulin;TG,triglycerides;FT3,free triiodothyronine;FT4,free thyroxine;TSH,thyroid- stimulating hormone.

**Table S2** Multiple linear regression analyses for association between Z-standardized TT4RI and lnHOMA-IR

| Model | Adjustment variables | β（95%CI） | *P* |
| --- | --- | --- | --- |
| Model 1 | Unadjusted | 0.079(-0.010,0.167) | 0.081 |
| Model 2 | Age,BMI | 0.040(-0.038,0.118) | 0.313 |
| Model 3 | Age,BMI,SBP,SHBG | 0.038(-0.034,0.110) | 0.299 |

Multiple linear regression analyses of the association between Z-standardized TT4RI and lnHOMA-IR. Model 1: unadjusted. Model 2: adjusted for age and BMI. Model 3: adjusted for age, BMI, systolic blood pressure (SBP), and sex hormone-binding globulin (SHBG). β, unstandardized regression coefficient; 95% CI, 95% confidence interval.
